# Qualitative Study on the Voices of Adolescents Living with Perinatally Acquired HIV in Selected Clinics in the Limpopo Province of South Africa

**DOI:** 10.3390/children11010028

**Published:** 2023-12-26

**Authors:** Rirhandzu Austice Mabasa, Livhuwani Muthelo, Linda Skaal, Tebogo Maria Mothiba

**Affiliations:** 1 Department of Optometry, Faculty of Health Sciences, University of Limpopo, Polokwane 0700, South Africa; tebogo.mothiba@ul.ac.za; 2 Department of Nursing Science, Faculty of Health Sciences, University of Limpopo, Polokwane 0700, South Africa; livhuwani.muthelo@ul.ac.za; 3 Department of Public Health, Faculty of Health Sciences, Sefako Makgato University, Ga-Rankuwa 0208, South Africa; linda.skaal@smu.ac.za

**Keywords:** disclosure, perinatally acquired HIV, adolescent voices, rural Vhembe district

## Abstract

The disclosure of HIV status among adolescents living with perinatally acquired HIV (APHIV) has become one of the core challenges in the management of APHIV. Disclosure is a challenge that undermines positive advances and achievements in HIV management. There is limited literature on the voices of APHIV on disclosure of their status. This study aims to explore the current disclosure process and how it affects APHIV. A qualitative exploratory design was employed to conduct one-on-one in-depth interviews using a semi-structured interview guide. Purposive sampling was used to sample 21 APHIV in 16 selected health facilities in the Vhembe district of Limpopo Province, South Africa. Data were analyzed using Tesch’s qualitative data method. The findings of this study reflect the gaps in the current disclosure process and guidelines while acknowledging the importance of disclosure to APHIV. A notable finding in this study is that most APHIV, especially those in early adolescence, did not want to know their HIV status due to the stigma attached to an HIV-positive diagnosis. This study suggests that proper training and support of parents and/or guardians in the disclosure process are needed, as they are the primary caregivers of APHIV. The disclosure of HIV status must be a comprehensive part of the management and care of HIV for APHIV. Furthermore, dedicated support programs should be developed and implemented to improve their lives post-disclosure.

## 1. Introduction

The current global estimate of adolescents aged 10 to 19 years who live with HIV is 1.7 million, of whom 85% reside in sub-Saharan Africa (SSA) [1]. The majority of these adolescents acquired HIV vertically [1,2]. It is unclear how many adolescents live with perinatally acquired HIV globally; however, this is an evolving and growing population as those who were not diagnosed as children are being diagnosed later in life [3,4,5].

According to the WHO, APHIV refers to adolescents who acquire HIV during conception, at birth, or during breastfeeding [6]. However, these adolescents were not disclosed during diagnosis due to their young age and the nature of acquisition. Their status was only disclosed later in life, leading to adverse health outcomes and other problems [1,7].

The World Health Organization (WHO) developed standard guidelines to be followed during the disclosure process [6,8]. Studies have recorded success stories resulting from the accurate application of the disclosure process, and most APHIV benefited from the disclosure process through empowerment and sustenance, as well as guidance on adherence to antiretroviral therapy (ART) [1,6]. However, there are unique issues in the current disclosure process that are not addressed by the guidelines and therefore undermine the main objective of disclosure [9,10]. The success of the disclosure process is determined by the reported positive coping skills by APHIV. However, few studies have examined the after-effects of disclosure among APHIV, leaving a gap in knowledge about the post-disclosure experiences of APHIV [11].

The literature shows a significant association between the disclosure of HIV-positive status in adolescents and decreased adherence [1,12]. Studies reported major health problems after disclosure, such as high rates of mental health vulnerabilities (e.g., maladaptive coping mechanisms) and high-risk behaviors such as alcohol and substance abuse and risky sexual behavior [13,14,15]. Few studies have explored the experiences of APHIV on current disclosure practices both before and after disclosure [9,16,17,18]. They are at the receiving end of the disclosure process, making their experiences vital to the effectiveness of the process. As mentioned above, there are reports of positive outcomes of the disclosure process among APHIV [19,20]. However, the unaddressed issues in this process undermine the positive effects of disclosure due to a high rate of mental health vulnerabilities (e.g., maladaptive coping mechanisms) and a high rate of non-adherence to ART post-disclosure. The nature of the acquisition of the disease among APHIV complicates the disclosure process, contributing to its failure [21,22].

There is limited literature on APHIV voices on their experiences with the current disclosure process and post-disclosure follow-up among APHIV. This study aims to explore the experiences of APHIV with current disclosure practices to inform the development of improved intervention strategies in HIV management.

## 2. Materials and Methods

A qualitative exploratory design was used to conduct in-depth interviews in the Vhembe district of Limpopo Province in South Africa at selected community health centers and clinics. This design aimed to qualitatively explore the challenges experienced by APHIV concerning the current disclosure process using life course theory and to determine the extent of the challenges faced by adolescents living with perinatally acquired HIV. According to Elder et al., the theory of life course refers to the approach to analyzing people’s lives within the structural, social, and cultural context [23].

### 2.1. Ethical Approval and Informed Consent

Permission to conduct the study was obtained from the Turfloop Research Ethics Committee (TREC/228/2019: PG) and the Limpopo Province Department of Health. A letter explaining what the study was about, including audio recordings of the interviews, was issued to all participants over 18 years of age and parents/guardians of APHIV under 18 years of age to obtain informed consent, as well as verbal consent from those under 18 years of age. Participants were also informed of their right to withdraw their participation at any point during the study.

### 2.2. Study Setting

The study was carried out in 16 health facilities within the Vhembe District Municipality of Limpopo Province in South Africa. The Vhembe district has few resources and is primarily rural. It has six district hospitals, one regional hospital, and one specialized hospital. The district also has 115 clinics, eight community health centers (CHCs), and 19 mobile clinics that are used to reach remote areas. The study setting consisted of 16 facilities, including three CHCs and 13 clinics, which were randomly selected. All selected facilities offer ART to children and adults and depend on nurses who were trained to initiate and manage ART through nurse-initiated antiretroviral therapy (NIMART).

### 2.3. Population and Sampling

The study population comprised 2117 APHIV in the Vhembe district. The researchers examined the eligibility of the HIV registry. Purpose sampling was used to select APHIV who had started ART before the age of 10. The APHIV who met the criteria were then recruited telephonically through their caregivers with the help of the staff working with APHIV, and interviews were then conducted. 

APHIV over 18 years of age received a letter that contained all the information about the study, as well as the aim and objectives of the study, to obtain informed consent. Those between 12 and 17 years of age were recruited through their caregivers and verbal consent was sought in the presence of their caregivers, who signed the consent form on their behalf. The objectives of the study were explained by the researchers before signing the consent form. A sample size of 21 APHIV of all genders were interviewed. Data saturation was attained at 21 where further sampling yielded similar results. All recruited APHIV agreed to participate; however, some did not honor their appointment dates, and others were excluded due to the attainment of data saturation. The sample was fully representative of APHIV in the Vhembe district and provided valuable information on the research topic. Participants were not reimbursed for their participation and interviews were held the day they were scheduled to attend the clinic.

### 2.4. Data Collection

The recruitment of APHIV was carried out telephonically and face-to-face for those who came to collect their medication at the clinic. For those who were recruited by telephone, appointments were arranged for face-to-face meetings. Consent was sought and interviews were conducted on the same day with those who were coming to collect medication. During face-to-face meetings, researchers briefed participants and caregivers on the study. APHIV who were 18 years and older signed an informed consent form and the caregivers of those between the ages of 12 and 17 years of age signed consent on their behalf. Data was collected using an in-depth interview guide. The researchers conducted one-on-one interviews with the help of two trained research assistants. The interviews were conducted in Xitsonga and Tshivenda, which are the two most spoken languages in the Vhembe district. Interviews were conducted for those between the ages of 12 and 17 years to allow them to speak freely. The interviews were conducted in a private consultation that was not frequently used and had minimal noise levels. The interviews were conducted for a duration of between 15 and 60 min each. Table 1 presents the interview questions below. This paper is part of a thesis; therefore, only participants who were aware of their HIV status were asked these questions to explore their experiences on the disclosure process. 

### 2.5. Data Analysis

The data was analyzed using the Tesch method of qualitative data analysis. The audio-taped data was transcribed and translated into English for analysis. Data were analyzed manually by reading all transcripts and writing ideas in the margins of each transcript. This helped the researchers make sense of the collected information. A list of all topics was divided based on similar topics and further divided into major topics, unique topics, or miscellaneous. The topics that emerged were organized into codes that were given the best wording for the topic. The total list of topics was reduced and turned into categories by grouping the topics related to each other. Categories were abbreviated and arranged alphabetically. Introductory analysis was carried out by collecting data material belonging to each category into one. Themes and sub-themes were summarized and sent to the independent coder. The final themes and subthemes were independently identified by the researchers and the independent coder.

## 3. Results

### 3.1. Description of the Study Sample

One-on-one interviews were conducted with 21 participants, 17 of whom were girls and who predominantly resided in rural areas. Most of the participants were between the ages of 16 and 19 and were in secondary school. Most of them had been on ART for more than 5 years. Eight participants had lost both parents and were under the care of relatives and/or guardians. Table 2 summarizes the demographic information of the participants. 

### 3.2. Themes and Sub-Themes

Themes and subthemes are presented in Table 3. The three themes that emerged are HIV-positive status disclosure, incorrect HIV status disclosure, and post-disclosure follow-up. Direct quotes from participants are presented in italics.

#### 3.2.1. Lack of HIV-Positive Status Disclosure

According to the WHO definition of disclosure, APHIV must be fully aware of their HIV status by the age of 12 [5]. This study found that none of the participating APHIV were disclosed by the age of 12 years, and the majority were disclosed later in life. Furthermore, three participants were unaware of their HIV status, even though they were taking medication on their own and were over the age of 12 years.

When Participant 008 was asked if she knew what medication she was taking, she replied: “I don’t know what this medication is for but I’m not sick, my grandmother told me that I must take this medication to prevent sickness”.

Two other participants gave similar responses when asked if they knew what drug they were taking and they said: “I don’t know”.

#### 3.2.2. APHIV Discovering Their Own HIV Status

Since there was a lack of disclosure, some APHIV discovered their HIV status on their own in their mid and late teens. One participant shared that she discovered her status by going through her clinic file and finding the letters “RVD” (retroviral disease) written in the file. Others said they found out by searching for their medication on the Internet.

Participant 006 said: “No one told me, I just discovered along the way that I am HIV positive when I was taking treatment. I read a lot and I read anything. I became curious when I had to take medication non-stop and no one told me what the medication was for. I searched for the name of the drug on the label and found that the drug was for HIV, so that is how I discovered my HIV status”.

Another participant said: “I was never told what the medication is for, but when I read my file it is written RVD. I asked one of the nurses what RVD means and she refused to tell me until I googled it and found that I live with HIV. I have not asked my mother about it; I think she will tell me when she is comfortable”.

“I grew up taking treatment, but I did not ask what the medication was for, because my grandmother gave it to me until 2017 when I fell pregnant, that’s when the nurses told me that I was born with HIV, so I have been taking this treatment since birth, so I had to continue taking my medication to prevent my child also from being born with HIV”.

#### 3.2.3. Fear of Knowing Their HIV Status

The findings show that APHIV are afraid of being diagnosed with HIV due to fear of the stigma attached to the diagnosis of HIV. Some indicated that they were comfortable with not knowing their diagnosis rather than being told that they have HIV. Although others were disclosed, they were not comfortable releasing them to researchers due to denial of their status.

Participant 011, aged 15 years old, said: “I grew up taking treatment and I am fine not knowing what it is for, as long as it keeps me well. I just don’t want to have HIV; I prefer to take my medication without knowing what it is for than to be told that I have HIV”. When asked why HIV was not detected since he did not know his diagnosis, he said, “HIV is the only disease I don’t want to have, I don’t want to be labelled and given ‘strange names’ by the community”.

Participant 001 said: “This disease of HIV that I have, I only heard people talking, saying HIV, HIV”.

### 3.3. Theme 2: Incorrect HIV Status Disclosure Process

#### 3.3.1. Informing Versus Disclosure: Lack of Proper Disclosure

APHIV was not properly disclosed. They were only informed of their HIV status. The findings showed that the disclosure of HIV status was often circumstantial. For example, APHIV were informed that they lived with HIV to prevent them from engaging in sexual relationships when caregivers felt that they were at risk of doing so.

Participant 004 said: “My mother gave me medication since I was young, but she never told me what the medication was for. One day when I turned 13 years old I started menstruating and I told my mother about it because we learnt at school that when we see blood, we must tell our parents. Days later she took me to the clinic and I was told that I was born HIV positive and that I live with HIV”.

“I found out in 2016 just after my parents passed away. My sister told me that my parents died of AIDS and passed away and I acquired it during birth, she explained that I was born with HIV and I have to take my medication daily for the rest of my life”.

#### 3.3.2. Incorrect/Wrong Diagnosis

The information provided by APHIV revealed that the disclosure process was not followed correctly. Parents/caregivers gave incorrect information about their diagnosis to avoid being asked questions. They received incorrect information to gain cooperation about treatment adherence.

Participant 005 said: “I grew up a sick child, so I was given medication for my sickness and I never questioned my medication because I thought it was for the sickness I had. As I grew older, when I asked why I was taking the medication even when I was not feeling sick, my grandmother only told me to take the medication so that I do not get sick and die like my mother”.

Participant 00 said: “I got tired of taking medication every day, since I didn’t feel sick. I sometimes refused to take medication and that is when my father told me that I had chronic tonsillitis and had to take the medication not to complicate and end up dead. I only discovered when I was growing up that the medication is not for tonsils as I was told and that I have AIDS”.

Another participant said: “My grandmother gave me the medication, I started to be curious as I grew up and wondered why I am the only one taking this medication. One day I got the strength and asked her what the medication was for, and she said that this medication was to remove my dust from my chest to prevent tuberculosis and that if I don’t take this medication, I will die”.

Other guardians instilled fear of death to force adherence to ART instead of revealing HIV status to APHIV in their care.

A participant said, “My grandmother told me to take the medication so that I don’t get sick and die like my mother”.

#### 3.3.3. Lack of Involvement in the Disclosure Process by APHIV

The information provided by the APHIV indicated that they were not involved in the disclosure process. They were not included in the decision on when and how they wanted to be disclosed. Instead, it was the parents, caregivers, and/or health workers who were involved in deciding how and when they were disclosed.

Participant 004 said: “My mom used to collect medication for me every month; I only went to the clinic for my blood once every 6 months. But the time she wanted to tell me that I live with HIV, she told me that I had to go to the clinic with her, and I had had my blood collected, so I was surprised”.

Participant 001 said: “My father told me that the nurses at the clinic wanted to see me and when I asked why, he said that he had no idea. When I got to the clinic with my father, they told me that I am HIV positive and that is why I am taking this medication”.

### 3.4. Post-Disclosure Process

#### The Role of Parents/Caregivers in the Disclosure Process

Almost all APHIV that were disclosed were told in their mid or late adolescence. Parents/caregivers decide when APHIV should be informed of their HIV status.

Participant 00 said: “My mother told me my HIV status when I was in grade 10, I was 16 years old then”.

Another participant said: “I only learnt about my HIV status last year when I was 17 years old, my mother told me that she always wanted to tell me my HIV status, however, she felt that I was still young and would not understand, that is the reason why she delayed the disclosure”.

## 4. Discussion

The development of disclosure guidelines was part of the strategies for the realization of SDG 3 by ensuring healthy lives and promoting well-being for all, at all ages [24,25]. They were developed as a standard guideline for healthcare workers for the provision of standard and quality health care to APHIV and caregivers. This study explored the experiences of APHIV on current disclosure practices in rural settings. The study findings noted that there were challenges in the implementation of the disclosure guidelines set out by the WHO. Disclosure of HIV-positive status in APHIV is a vital tool in the achievement of the SDG.

### 4.1. HIV-Positive Status Disclosure Process

APHIV shared their experiences regarding the disclosure of their HIV status. They shared varied experiences on how they were disclosed and their experiences post-disclosure. The results found that most APHIV were not happy with their disclosure experiences; there were errors in current disclosure practices that affected their journey to live with HIV. Although none of them reported major mental health effects post-disclosure, they mentioned how they found out their HIV status was traumatic and affected them emotionally. For example, most cited that they felt angry and had many unanswered questions after disclosure. These findings are consistent with previous studies indicating that APHIV which was not properly disclosed found the disclosure process to be traumatic and experienced emotional and psychological turmoil as a result [16].

The main purpose of the disclosure is to allow APHIV to have full autonomy in their lives and to empower them to navigate living with HIV. This was corroborated by a study conducted by Kitetele et al. (2023), who found that APHIV that were fully disclosed felt like their HIV-negative peers when they had to make informed decisions about sexual relationships [16]. Furthermore, different authors acknowledge the positive association between full disclosure and better adherence to ART, participation in less risky lifestyles, and improved mental health [1,7,19,26].

The theory of life course recognizes that people are capable of making choices if they are provided with information [23]. The current study found that some APHIV were not aware of their HIV status, as they were not disclosed to their parents/guardians. According to the WHO, the disclosure process should begin at the age of 6 and be completed at the age of 12 [1,10,16,17]. Studies in South Africa (SA) and other parts of the world report a low disclosure rate, especially in rural settings as a result of illiteracy among caregivers [17,19]. The findings of this study further highlight the added challenges faced by APHIV in rural areas where cultural values and norms affect disclosure practices.

Other studies have found that parents/caregivers delayed the disclosure of APHIV because they believed that the disclosure could have a negative psychological effect on children and that they would not be able to keep the secret, resulting in stigmatization [18,27,28]. The information provided by the participants highlighted that delays in disclosure can result in erroneous disclosure of HIV status to APHIV. For example, most APHIV were disclosed circumstantially due to parents’ fear of early sexual debut and teenage pregnancy. The delay further resulted in malicious disclosure to APHIV. The delay, according to other studies, was due to the fear of divulging their own HIV status to APHIV [29]. Participants cited that they learned about their HIV-positive status when their guardians revealed their HIV status to other people without their consent. A study in Ethiopia found that delayed disclosure resulted in APHIV being maliciously or spitefully disclosed by guardians after non-disclosure by parents [28]. The current study and other studies acknowledged that malicious disclosure caused emotional challenges, as APHIV cited that they were furious for a while and felt anxious and depressed [27].

The current findings showed that APHIV were given a wrong diagnosis that is not stigmatized. For example, some were told that they were taking medication for tonsillitis or to clear their chest. A study by Molokwane and Madiba found that parents lied about the diagnosis of APHIV or used coercion and threats to enforce treatment adherence and avoid disclosure [27]. The current study suggests incorporating the disclosure process into routine care and management of APHIV to alleviate the burden of parents revealing their HIV status. Furthermore, decentralization of HIV should start in the health facility by treating HIV like any other chronic disease which does not require a special disclosure process.

### 4.2. Challenges with the Disclosure Process

This study shows that the disclosure process in most households is initiated by parents/caregivers; they determine the right time to disclose to APHIV. This is consistent with the findings of other scholars who raised concerns that, despite the age of the APHIV, the decision to initiate the disclosure was unilateral; APHIV were excluded from the decision-making process, which undermines the positive effects of the disclosure process [22,27]. The findings highlight the impact of an improper disclosure process on APHIV mental health. APHIV raised concerns that they were informed about their HIV status without prior preparation, leaving them to deal with the aftereffects of the disclosure without the necessary support. Studies in SSA raised concerns that current health policy has neglected the special needs of APHIV, including their mental health, thereby undermining the positive milestones made in their care and management [24,28,29,30].

The current study found that the disclosure process was initiated in the mid and late teens of APHIV’s lives. These findings are consistent with other studies around the world, some of which established that disclosure was based on the age and maturity of APHIV to be able to handle the psychological impact of their HIV status and their ability to keep their HIV status a secret [27,31]. Other studies noted that delay in disclosure contributes to non adherence to ART, as well as other emotional challenges [18].

A notable finding in this study is that most APHIV, especially those in early adolescence, did not want to know their HIV status due to the stigma attached to an HIV-positive diagnosis. The experiences shared by APHIV showed that they preferred to take medication without knowing about their diagnosis, as they did not want to be told that they were living with HIV. These results are in contrast to other studies that found that APHIV preferred to be disclosed to [16]. The literature noted the negative mental and psychosocial effects of unprepared disclosure [16,17,18,32]. The findings of the current study highlight the psychological impact that incorrect disclosure might have on APHIV.

## 5. Limitations of the Study

As this study was conducted in one rural district of Limpopo Province, the results cannot be generalized to other settings, especially urban settings. Some APHIV were unable to articulate themselves well due to their young age and the secrecy surrounding HIV in their communities. However, the recommendations can be applied in other parts of South Africa.

## 6. Contribution to the Body of Knowledge

This study presents the post-disclosure experiences of APHIV in a rural context, a population whose experiences are usually not documented. This suggests that context should be considered when evaluating the disclosure processes and guidelines currently in place.

This study also builds a basis for future research on strategies to improve the lives of APHIV by making changes to the existing disclosure process and ensuring that it is followed correctly by parents, guardians, caregivers, and healthcare workers who are tasked with caring for APHIV.

## 7. Conclusions

The findings of this study reflect the gaps in the current disclosure process and guidelines while acknowledging the importance of disclosure to APHIV. The disclosure process should therefore be a multidisciplinary process involving supporting staff, such as home-based caregivers. The Department of Health (DOH) should involve other departments, such as the Department of Social Development (DSD), to assist with post-disclosure counseling. Scheduled compulsory classes for APHIV and caregivers could enhance the effectiveness of the disclosure process. There is a need for a different approach to implementing current WHO-recommended disclosure guidelines and processes, specifically the active participation of APHIV and caregivers alongside healthcare workers. Empowering parents, guardians, and caregivers of APHIV and strengthening the benefits of following the correct disclosure process and guidelines are vital to implementing an effective disclosure process.

## Figures and Tables

**Table 1:** Interview guide.

Central question Could you kindly describe your experience when you discovered your condition/status?
Probing Questions
How did you learn about your status? At what age did you learn about your status?
Can you kindly walk me through the process when you were informed/told about your status? Kindly describe your reaction after discovering your status. What kind of support did you receive after learning about your condition?

**Table 2:** Demographic information of the participants.

Item		Number
Place of stay	Rural	12
	Semi-urban	5
	Urban	4
Education level	Primary	5
	Secondary	16
Years on ART	Under 5 years	0
	Above 5 years	21
Years of orphanage	Under 5 years	2
	Above 5 years	6
Disclosure status	Disclosed	18
	Not disclosed	3
Who do they live with?	Both parents	3
	One parent	10
	Relatives or guardians	8

**Table 3:** Themes and sub-themes.

HIV-positive status disclosure process to APHIV	Lack of disclosure of HIV status
	Discovery of your own HIV status by APHIV
	Fear of knowing your own HIV status
Incorrect HIV status disclosure process	Informing versus. disclosure of HIV status
	Incorrect diagnosis
	Lack of involvement of APHIV in the disclosure process
Post-disclosure	Caregiver/parent role in the disclosure process
	Lack of follow-up

## Data Availability

The data presented in this study are available on request from the corresponding author. The data are not publicly available to protect the privacy of the participants.
